# Cross-Continental Dispersal of Major HIV-1 CRF01_AE Clusters in China

**DOI:** 10.3389/fmicb.2020.00061

**Published:** 2020-01-31

**Authors:** Minghui An, Xiaoxu Han, Bin Zhao, Suzanne English, Simon D. W. Frost, Hongyi Zhang, Hong Shang

**Affiliations:** ^1^NHC Key Laboratory of AIDS Immunology (China Medical University), Department of Laboratory Medicine, The First Affiliated Hospital of China Medical University, Shenyang, China; ^2^National Clinical Research Center for Laboratory Medicine, The First Affiliated Hospital of China Medical University, Shenyang, China; ^3^Key Laboratory of AIDS Immunology of Liaoning Province, The First Affiliated Hospital of China Medical University, Shenyang, China; ^4^Key Laboratory of AIDS Immunology, Chinese Academy of Medical Sciences, Shenyang, China; ^5^PHE Clinical Microbiology and Public Health Laboratory, Addenbrooke’s Hospital, Cambridge, United Kingdom; ^6^Department of Veterinary Medicine, University of Cambridge, Cambridge, United Kingdom

**Keywords:** HIV-1, CRF01_AE, cross-continental, dispersal, China

## Abstract

Since the 1990s, several distinct clusters of human immunodeficiency virus-type 1 (HIV-1) CRF01_AE related to a large epidemic in China have been identified, but it is yet poorly understood whether its transmission has dispersed globally. We aimed to characterize and quantify the genetic relationship of HIV-1 CRF01_AEs circulating in China and other countries. Using representative sequences of Chinese clusters as queries, all relevant CRF01_AE pol sequences in two large databases (the Los Alamos HIV sequence database and the UK HIV Drug Resistance Database) were selected with the online basic local alignment search (BLAST) tool. Phylogenetic and phylogeographic analyses were then carried out to characterize possible linkage of CRF01_AE strains between China and the rest of the world. We identified that 269 strains isolated in other parts of the world were associated with five major Chinese CRF01_AE clusters. 80.7% were located within CN.01AE.HST/IDU-2, most of which were born in Southeast Asia. 17.8% were clustered with CN.01AE.MSM-4 and -5. Two distinct sub-clusters associated with Chinese men who have sex with men (MSM) emerged in HK-United Kingdom and Japan after 2000. Our analysis suggests that HIV-1 CRF01_AE strains related to viral transmission in China were initially brought to the United Kingdom or other countries during the 1990s by Asian immigrants or returning international tourists from Southeast Asia, and then after having circulated among MSM in China for several years, these Chinese strains dispersed outside again, possibly through MSM network. This study provided evidence of regional and global dispersal of Chinese CRF01_AE strains. It would also help understand the global landscape of HIV epidemic associated with CRF01_AE transmission and highlight the need for further international collaborative study in this field.

## Introduction

The HIV-1 pandemic is currently dominated by Group M, which has diversified into nine subtypes, seven sub-subtypes (A1, A2, A3, A4, A6, F1, and F2), 98 circulating recombinant forms (CRF) and numerous unique recombinant forms (URF). Human migration, globalization, and different risk factors for transmission between hosts have shaped the geographical and demographic distribution of HIV. For example, strains of CRF01_AE and B co-circulate among high risk sexual population and injecting drug users (IDUs) in East and South-east Asia, in contrast to MSM population in Western Europe and North America, where circulating HIV-1 strains are dominated by subtype B (Peeters et al., 2013).

However, recent studies have reported the emergence and rapid increase of non-B subtypes in various populations globally (Cuevas et al., 2009; Liao et al., 2009; Hawke et al., 2013; Neogi et al., 2014; Beloukas et al., 2016; Nikolopoulos et al., 2016; Dennis et al., 2017), especially among men who have sex with men (MSM) in China (Zhang et al., 2015). CRF01_AE, CRF_BC and Subtype B/B’ are most prevalent in Mainland China (Su et al., 2014), and CRF01_AE has recently replaced subtype B as the dominant circulating subtype among Chinese MSM (Zhang et al., 2015). A number of detailed phylogenetic analyses revealed that several major transmission clusters of HIV-1 CRF01_AE existed and expanded after their multiple introductions into China (Abubakar et al., 2013; Feng et al., 2013; Peng et al., 2015; Zeng et al., 2016; Wang et al., 2017). Three of them were prevalent among heterosexuals and IDUs (CN.01AE.HST/IDU-1/2/3 clusters), and two were related to MSM transmission (CN.01AE.MSM-4/5 clusters) (Feng et al., 2013). By contrast, in Western Europe and North America, subtype B was the early epidemic strain and now is still dominating (Hemelaar, 2013). However, some studies also reported that the frequency of non-B subtypes has been increasing in Western Europe and North America (Thomson and Najera, 2007; UK Collaborative Group on HIV Drug Resistance, 2014), especially among MSM population of eight countries in North America, Western Europe and Australia (Sullivan et al., 2009). Recent studies of European HIV populations have reported that CRF01_AE strains have emerged in European MSM population, accounting for 11.2% in the United Kingdom (Fox et al., 2010) and 11.1% in Spain (Cuevas et al., 2009), among newly detected non-B strains. However, few studies have focused on the genetic links of HIV-1 CRF01_AE strains between countries or regions.

While several distinct clusters of CRF01_AE have been identified to be related to a large epidemic in Mainland China, it is poorly understood whether it has dispersed elsewhere. Therefore, the aim of this study was to characterize and quantify the genetic relationship of Chinese HIV-1 CRF01_AE strains with those circulating in other countries or regions globally. In particular, we focused on the linkage between CRF01_AE strains circulating among Chinese and United Kingdom populations, due to the availability of a large, diverse sequence database available in the United Kingdom (UK Collaborative HIV Cohort Steering Committee, 2004).

## Materials and Methods

### Sequence Databases

HIV-1 CRF01_AE sequences covering the protease and partial reverse transcriptase coding regions (∼1.0 kb) were acquired from a public database, the Los Alamos HIV Sequence Database^1^, and the UK HIV Drug Resistance Database (UKHDRD)^2^ which is a centralized database of pol gene fragments generated from plasma samples collected throughout the United Kingdom as part of routine clinical care, largely during drug-naive chronic infection but also during acute infection and antiretroviral therapy failure. Sequence data are linked to demographic and clinical patient data held by the United Kingdom Collaborative HIV Cohort study (UK Collaborative HIV Cohort Steering Committee, 2004) and the national HIV/AIDS Reporting System database held at Public Health England (Public Health English, 2008). The London Multicenter Research Ethics Committee approved the use of the anonymous data in the UKHDRD. This collaborative project and its access to the database was approved by the UKHDRD Steering Committee.

### Query Sequences for Initial Blast Search

**FIGURE 1:**
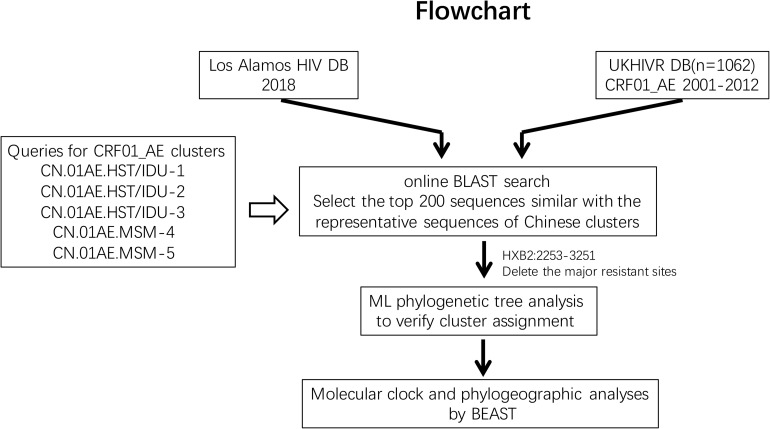
The study flowchart used to screen and identify HIV-1 CRF01_AE strains associated with viral transmission in China. The Blast tool was used to search the sequence of interest in (i) the Los Alamos HIV sequence database and (ii) the United Kingdom HIV Drug Resistance Database. Five published Chinese major CRF01_AE clusters were used as queries (covering protease and partial reverse transcriptase nucleotide sequences). The top 200 sequences were selected and included in this analysis according to Blast scores. Phylogenetic and phylogeographic analyses were reconstructed to identify the relationship between Chinese CRF01_AE and CRF01_AE from other countries/regions globally.

The initial searching procedures used to screen and identify HIV-1 CRF01_AE strains associated with viral transmission in China from the above two databases are shown in Figure 1. Five predominant CRF01_AE clusters in China have been described previously (Feng et al., 2013). In order to make the query sequence more representative and avoid any potential bias due to genetically similar queries, three query sequences from three different regions, sampled at different collection dates and showing the largest genetic distance between each other within one cluster were selected for each of the five clusters. These 15 query sequences are as follows: FJ070010 (accession number JX112809), GD070010 (JX112819), and GX070003 (JX112829) for CN.01AE.HST/IDU-1 cluster; FJ070013 (JX112810), GX070005(JX112830), and JS070901 (JX112850) for CN.01AE.HST/IDU-2 cluster; GD070120 (JX112827), GZ070123 (JX112843), and FJ052 (EF036528) for CN.01AE.HST/IDU-3 cluster; CYM059 (JX112796), TJ070003(JX112859), and JS071101 (JX112853) for CN.01AE.MSM-4 cluster; CYM105 (JX112798), JL100005 (JX112846), and LN070008 (JX112854) for CN.01AE.MSM-5 cluster. An approximately 1.0 kb fragment from nucleotide 2253 to 3251 (corresponding to HIV-1 HXB2 sequence) in the pro-RT coding region of each query sequence was used for the Blast search.

### Initial Blast Search and Related Sequence Identification

The online basic local alignment search tool (BLAST)^3^ was used to search the above two databases for all sequences which match the pro-RT fragment of any query sequence. For each Chinese CRF01_AE cluster, the first 200 sequences with the highest Blast score were selected from each of three query sequences. A total of 6000 sequences from two databases were selected for the five major CRF01_AE clusters. If there are duplicate sequences from an infected individual, only one of these sequences from this individual was retained for analysis. As recombination can affect the phylogenetic inference, so the recombinant signals of all sequences were analyzed in Recombination Detection Program 4 (RPD4) (Martin and Rybicki, 2000) before the reconstruction of phylogenetic tree. After removal of duplicates and recombinants, the retained sequences were used in phylogenetic analysis.

### Reconstruction of Phylogenetic Trees

We reconstructed the phylogenetic relationship to confirm possible links between global sequences and Chinese CRF01_AE clusters. We also downloaded some reference sequences most of that were sampled during 1990s from the Los Alamos HIV sequence database, representing the primary geographical spread of CRF01_AE as follows: Africa (4 Central African Republic, 2 Cameroon, 1 Congo, 1 Gabon, and 1 Senegal), Europe (5 Switzerland, 2 France, 2 Sweden, 1 Germany and 1 Denmark), America (3 United States), South-east Asia (15 Thailand and 1 Vietnam), to fix the topology of phylogenetic tree. The phylogenetic trees were rooted using an outlier group containing three subtype C strains. The retained sequences after initial Blast search and 42 reference sequences were codon-aligned using the Gene Cutter tool (see footnote 3) and then manually edited using BioEdit 7.2. To mitigate the effect of antiretroviral therapy-related selection pressure on the phylogenetic analysis, all major HIV-1 drug resistance mutation sites were deleted according to the 2019 update of the drug resistance mutations in HIV published by the international AIDS Society, United States. ModelFinder package of IQ-TREE was used to judge the best fitting nucleotide substitution models and the maximum-likelihood (ML) tree was finally reconstructed under the generalized time reversible model of nucleotide substitution with gamma distribution for rate heterogeneity (GTR + I + G) using IQ-TREE (version 1.6.12) (Nguyen et al., 2015). The branch support was estimated with the approximate likelihood-ratio (aLRT) SH-like test.

The access numbers of reference sequences downloaded from the Los Alamos HIV sequence database were as followed: AM040989, AF197340, AF197341, U51188, AY626951, GU207116, AM279456, AJ313417, AJ287011 in Africa, JF769840, KX691969, GQ848140, JF769833, GQ131600, AF347337, AM933273, AJ287043, AJ287053, AY165194, AY165218 in Europe, AY444803, AY444805, AY444806 in America, AF259954, U54771, AY713425, AF447828, AB220947, AF164485, AB220944, AB032741, AY713424, AY713419, AY125894, AY713422, AY945726, AY358040, AY945719, FJ185238 in South-east Asia, and AB023804, AB067155, AB254141 of subtype C.

### Molecular Clock and Phylogeographic Analyses

The viral spatiotemporal trajectories were estimated through the time to Most Recent Common Ancestor and the localization of ancestors, using a Bayesian Markov Chain Monte Carlo (MCMC)-approach (BEAST v1.10.4 package), under GTR + I + G nucleotide substitution model. The sequences with available sampling date and locations that were identified to be associated with viral transmission in Chinese major CRF01_AE clusters were used to reconstruct the maximum clade credibility (MCC) tree. The posterior distribution was tested under a relaxed lognormal molecular clock, which has previously been shown to be more reliable in estimating viral phylogenies and divergence dates than “strict clock” and “non-clock” methods (Drummond et al., 2006). Several demographic models (parameter models: constant, exponential and logistic non-parameter models: skyline, skyride and skygrid) were compared using marginal likelihood estimators based on path sampling and stepping-stone sampling (PS/SS) analysis in BEAST v1.10.4, which would make sure the best fit model for the demographic signal was used. The MCMC chains were run for at least 200 million times and sampled every 20000 steps, for several times until getting the good convergence status. The output was analyzed in Tracer (version 1.5) and the related parameter estimates with an Effective Sample Size (ESS) over 200 were accepted. After the initial trees were summarized by TreeAnnotator (with 10% burn-in), the MCC tree was visualized and color-annotated with the FigTree (version 1.3). The median tMRCA was reported along with 95% HPD intervals.

### Identification of Clusters

The transmission clusters were initially estimated using a maximum-likelihood approach. Potential clusters were pre-defined as three or more sequences together in the reconstructed topology of the phylogenetic tree with the aLRT statistical support value of >0.9. Subsequently, clusters were re-supported at a posterior probability of 1.0 by Bayesian phylogenetic inference.

## Results

### The Global Dispersal of HIV-1 CRF01-AE Strains Associated With the Transmission in Mainland China

**FIGURE 2:**
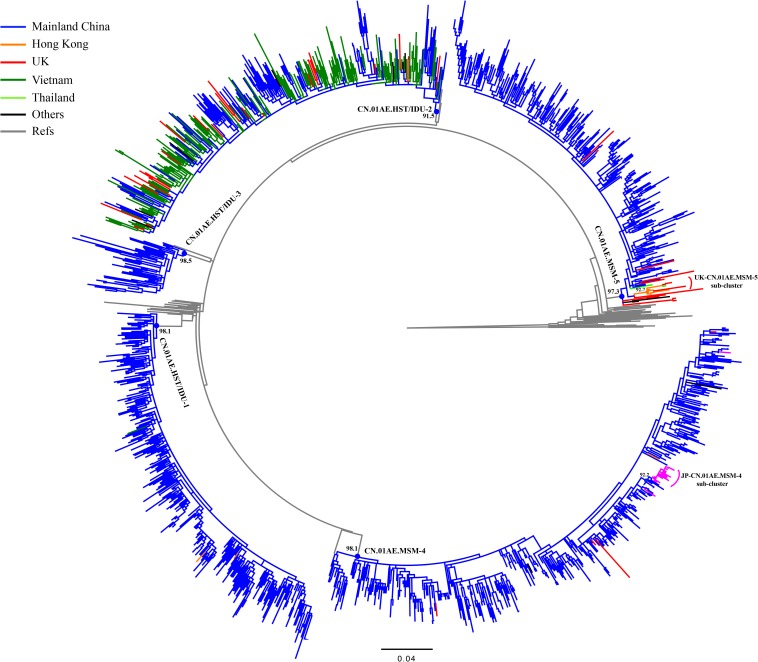
Phylogenetic analysis of global CRF01_AE strains outside Mainland China together with five major Chinese clusters. After the online BLAST of the two databases, the phylogenetic analysis of all selected sequences related to viral transmission of five major Chinese HIV-1 CRF01_AE clusters of interest was performed to demonstrate any possible linkages of CRF01_AE strains between China and the rest of the world. The maximum likelihood (ML) tree was reconstructed using the 1.1-kb pol sequences under GTR + I + G sites substitution model. The sample countries of each sequence are represented by different colors. The ML tree is rooted with three subtype C sequences as outgroup. The sequences that un-clustered with major five Chinese clusters were deleted in this reconstruction and the Supplementary Figure S1 showed the raw phylogenetic ML tree using the whole 2150 sequences in online BLAST search.

After the initial search using 15 representative query sequences (three per cluster) in two sequence databases, 3850 duplicate sequences were removed from initial 6000 blast results and a large number of unique sequences (*n* = 2150) of HIV-1 CRF01_AE were retained for phylogenetic analysis, of which 1202 were Chinese sequences and 948 were from elsewhere (Supplementary Data Sheets 1, 2). Then, 1167 sequences from Mainland China and 269 sequences from elsewhere were identified to be possibly associated with the HIV-1 CRF01-AE transmission clusters in Mainland China, based on the topology of ML tree and >0.9 branch support values (Supplementary Figure S1 and Figure 2). Of these 269 foreign sequences, 212 were Asian sequences (181 from Vietnam, 18 from Japan, 9 from Hong Kong, 2 from Thailand, 2 from Malaysia) and 55 were European (48 from the United Kingdom, 4 from the Czechia, 1 from Germany, 1 from Sweden, 1 from Russia), and 2 were Australian (Table 1).

**TABLE 1:** Distribution of non-Chinese CRF01_AE sequences associated with the major Chinese clusters in the searched databases.

Clusters	Risk group involved	Database searched	Sampling year									*n*	Total
			1997–2007	2008	2009	2010	2011	2012	2013	2014	2015		
CN.01AE.HST/IDU-1	Hetero/IDU	Los Alamos United Kingdom	1(HK)			1(HK)		1(VN)				3	3
												0	
CN.01AE.HST/IDU-2	Hetero/IDU	Los Alamos United Kingdom	1(AU),4(CZ),1(SE),124(VN)	1(RU),12(VN)	13(VN)		1(VN)	30(VN)				187	217
			13	9	4		3	1				30	
CN.01AE.HST/IDU-3	Hetero/IDU	Los Alamos United Kingdom		1(HK)								1	1
												0	
CN.01AE.MSM-4	MSM	Los Alamos United Kingdom	1(HK)		1(JP)	1(AU),6(JP)	5(JP)	3(JP)	1(DE),1(JP)	1(JP)	1(JP)	21	25
						1	2	1(VN)				4	
CN.01AE.MSM-5	MSM	Los Alamos United Kingdom	3(HK),1(TH)	2(HK)			1(MY)		1(TH)			9^#^	23
			2		3	3	3	3				14	

*The region or country of origin of the sequences in Los Alamos HIV sequence database is indicated: Australia (AU), Czechia (CZ), Sweden (SE), Vietnam (VN), Russian Federation (RU), Hong Kong (HK), Japan (JP), Thailand (TH) and Malaysia (MY). Five major Chinese CRF01_AE lineages were shown (Feng et al., 2013). For the Los Alamos HIV sequence database, we did not tabulate the sequences from Mainland China. NA, Not Applicable. ^#^Including one sequence with unknown sampling year from Malaysia.*

As shown in Table 1 and Figure 2, out of 269 sequences sampled outside Mainland China, 217 (80.7%) matched CN.01AE.HST/IDU-2 cluster with the branch value of 0.92, but did not form any obvious epidemic sub-clusters. These included 180 strains from Vietnam, 36 from Europe (30 in the United Kingdom, 4 in Czechia, 1 in Sweden and 1 in Russia) and 1 strain from Australia, and most of them were sampled before 2010. All 30 UK CRF01-AE samples in CN.01AE.HST/IDU-2 were from non-white ethnic subjects. Most of them were heterosexuals or IDUs (77%, 23/30), and were born in South or Southeast Asian countries (60%, 18/30).

A total of 48 (17.8%) of the 269 sequences sampled outside Mainland China, matched CN.01AE.MSM-4 and CN.01AE.MSM-5 clusters with the branch value of 0.98 and 0.97 (Figure 2). Within both of the two clusters circulating among MSM population in China, two distinct monophyletic sub-clusters were identified with good statistical support (SH-like value = 0.92 and 0.93, respectively) as shown in Figure 2. The sub-cluster located in CN.01AE.MSM-4 is consisted of 16 Japanese sequences sampled during 2009–2013, designated as JP-CN.01AE.MSM-4 sub-cluster (Figure 2). We noted that the remaining two sequences from Japan also belong to CN.01AE.MSM-4. Further, four United Kingdom sequences located within CN.01AE.MSM-4, and all of them were from MSM and two were born in East Asia. Another sub-cluster is within CN.01AE.MSM-5, consisted of three United Kingdom, five Hong Kong and one Thailand sequences, and because of the historical close link between United Kingdom and HK, we designated it as UK-CN.01AE.MSM-5 sub-cluster (Figure 2). The three United Kingdom sequences were isolated in 2011–2012 from white males (MSM), while the five HK sequences were isolated in 2007–2008. Notably, other six United Kingdom heterosexual sequences were placed at the root of CN.01AE.MSM-5, most of which were white males (heterosexuals).

Additionally, our analysis also revealed that a few sequences outside mainland China (three from HK and one from Vietnam) belonged to CN.01AE.HST/IDU-1 and 3, which were prevalent among heterosexuals and IDUs in China (Table 1 and Figure 2).

### The Spatial-Temporal Scale of HIV-1 CRF01-AE Strains Associated With the Transmission in Mainland China

**FIGURE 3:**
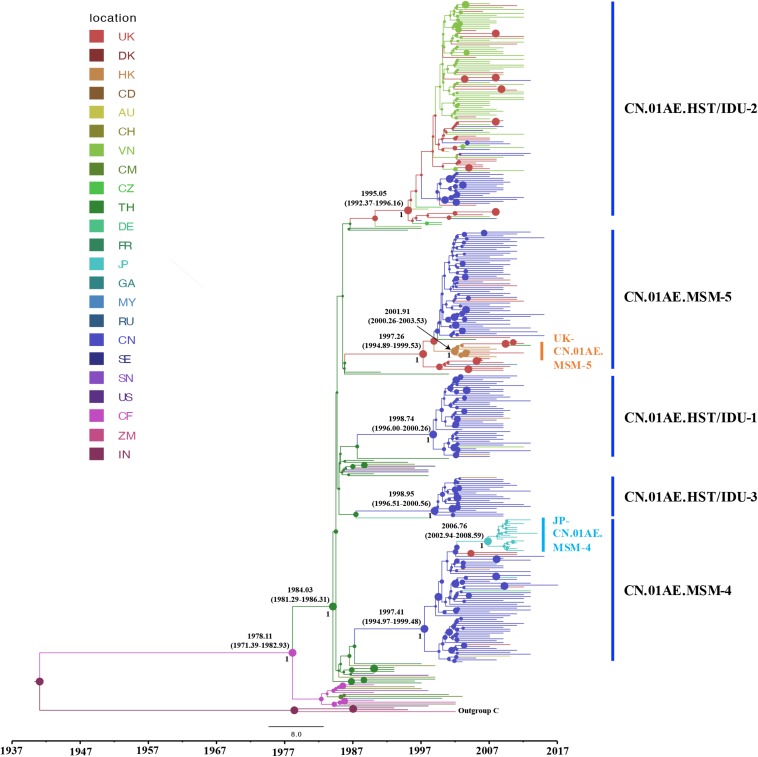
The origins and dispersal history of HIV-1 CRF01_AE associated with viral transmission in Mainland China. The maximum clade credibility (MCC) tree was reconstructed using the subsampled sequences (*n* = 328) in an online web server of CD-HIT-EST program, under GTR + I + G sites substitution model, the uncorrelated relaxed clock model and the Bayesian SkyGrid model through the Bayesian inference approach. The colors of nodes and branches represent the locations of the estimated ancestors for clades and the sampling locations of each sequence. The size of node represents the strength of node support (posterior probability). The tMRCAs of the representative clusters are indicated at the node.

To identify any export events of CRF01_AE strains related to Chinese major transmission clusters, we quantified the genetic divergence time in terms of the estimated time to the most recent common ancestor (tMRCA) using a Bayesian MCMC-based approach and also estimated the location of ancestor at the node of the tree. Many sequences (1172 from China and 226 from Vietnam) were identified to be associated with viral transmission in China through above phylogenetic analysis, and to reduce the computational burden in Bayesian running for spatial-temporal analysis, a sub-sampling procedure was performed that only one representative sequence was selected among high similarity clustered sequences (genetic distance < 2%), using the online web server of CD-HIT-EST program (Huang et al., 2010; Eybpoosh et al., 2017). As a result, totally 328 sequences (186 from Mainland China, 56 from Vietnam, 88 from other countries/regions) and 42 references were used for the phylogeographic reconstruction. For this dataset, the Bayesian SkyGrid model was identified as the best fit coalescent tree prior after the comparison of different demographic models by a Bayes Factor (BF), using marginal likelihood estimators based on PS/SS analysis in BEAST v1.10.4 (Supplementary Table S1). As shown in Figure 3, the estimated tMRCAs of CN.01AE.HST/IDU-1, CN.01AE.HST/IDU-2 and CN.01AE.HST/IDU-3 were 1998.74 (95% HPD interval 1996.00–2000.26), 1995.05 (1992.37–1996.16) and 1998.85 (1996.51–2000.56), respectively. The estimated tMRCAs of CN.01AE.MSM-4 and CN.01AE.MSM-5 were 1997.41 (1994.97–1999.48) and 1997.26 (1994.89–1999.53), respectively. The estimated tMRCAs of JP-CN.01AE.MSM-4 and UK-CN.01AE.MSM-5 sub-clusters were 2006.76 (2002.94–2008.59) and 2001.91 (2000.26–2003.53), respectively. Remarkably, six United Kingdom heterosexual strains and one Malaysian strain were seen at the root within CN.01AE.MSM-5, and the inner cluster of Chinese MSM strains was placed inside these six United Kingdom strains, supported with a posterior probability value of 0.89, and the estimated tMRCA of 1998.85.

For either ML tree or MCC tree, African strains were at the root and Thailand strains were highly centralized outside Chinese clusters, and European-American strains dispersed across the tree. The divergence times of African and Asian CRF01-AE strains were 1978.11 (1971.39–1982.93) and 1984.03 (1981.92–1986.31). This is in agreement with the hypothesis that HIV-1 CRF01_AE originated from Africa and then migrated to Thailand, from which, as the secondary source, the virus subsequently, spread to most areas worldwide (Angelis et al., 2015).

## Discussion

In recent years, HIV-1 non-B subtypes increased rapidly, not only in Asia, but also in Europe-America where HIV-1 subtype B is generally dominating. In Mainland China, HIV-1 CRF01_AE has caused a large epidemic, and several distinct clusters related to transmission among various high-risk populations have been identified. This study is the first detailed analysis of the possible dissemination of major HIV-1 CRF01_AE clusters from Mainland China to other countries or regions.

Angelis et al. (2015) reported that China was a sink and CRF01_AE strains were mainly imported from neighboring countries and then evolved into “distinct” Chinese strains, based on a global dataset of 2736 CRF01-AE sequences acquired from many public databases and cohort studies. Our results showed that although major HIV-1 CRF01_AE clusters in China were imported from Thailand, one of which might have entered China via Vietnam (CN.01AE.HST/IDU-2 cluster) (Figures 2, 3) (Liao et al., 2009), and these Chinese HIV-1 CRF01_AE strains had spread to other countries/regions.

After using 15 representative query sequences for initial blast screening and further phylogenetic analysis, 269 samples from other countries or regions matched the five major CRF01_AE clusters associated with HIV transmission in China. Among these, 80.7% (217/269) belong to CN.01AE.HST/IDU-2 cluster, most of which were sampled in Vietnam, and the rest from the United Kingdom or other countries. None of these 217 samples formed any distinct sub-clusters within CN.01AE.HST/IDU-2 cluster (Figures 2, 3), and most were sampled before 2010 (Table 1). Therein, of 30 individuals diagnosed in the United Kingdom as HIV-1, 18 are with a South/Southeast Asia origin and 23 were infected via heterosexual contact or drug injection (Table 2). The molecular clock analysis indicated that the tMRCA of CN.01AE.HST/IDU-2 was ∼1995, earlier than other four Chinese clusters (Figure 3). It is known, many Vietnamese started to immigrate to the United Kingdom since the end of Vietnam War in 1975, and because the Vietnamese communities in the United Kingdom were very small and closed, it was hard to integrate themselves further to the host communities (Fakoya et al., 2015; Vietnamese people in the United Kingdom). We infer that these HIV-1 CRF01_AE strains that own a common ancestor with Chinese CRF01_AE strains, circulating among Asian communities in Europe might be brought in by Vietnamese immigrants around mid-1990, and due to the closed feature of Vietnamese communities in the United Kingdom, the strains did not enter the local British communities. Similarly, six samples from the UK Drug Resistance Database were seen at the root of CN.01AE.MSM-5 with high support value (Figures 2, 3). Four of six were born in the United Kingdom and two were born in Southeast Asia, and all the six United Kingdom individuals were infected via heterosexual contact (Table 2). The divergence time of CN.01AE.MSM-5 was ∼1997 (Figure 3). Due to Thailand’s sex industry and the popular international tourism to Thailand during the last three decades (Angelis et al., 2015), the CRF01_AE strains associated with CN.01AE.MSM-5 might be brought back to the United Kingdom initially by returning tourists who were infected via heterosexual contact in Southeast Asia. Although the estimated locations of ancestors of CN.01AE.HST/IDU-2 and CN.01AE.MSM-5 were both in the United Kingdom as shown in phylogeographic tree (Figure 3), we inferred these United Kingdom individuals were most likely infected with Southeast Asian CRF01_AE, such as Thailand or Vietnam, and so the ancestors of these two clusters should still be located in Southeast Asia. It is therefore suggested that the CRF01_AE strains associated with viral transmission in China (CN.01AE.HST/IDU-2 and CN.01AE.MSM-5) were likely introduced to the United Kingdom initially by the Vietnamese immigrants and also returning tourists from Southeast Asia around the middle 1990, through the routes of heterosexual contact and/or drug injection. These CRF01_AE strains also spread to China around the same time from Southeast Asia and then formed two distinct clusters. Although Chinese sequences and foreign sequences within CN.01AE.HST/IDU-2 and CN.01AE.MSM-5 shared the same common ancestor, they did not have direct transmission relationship.

**TABLE 2:** Summary of epidemiologic and demographic information of subjects in the UK HIV Drug Resistance Database who were infected with HIV-1 CRF01_AE strains associated with Chinese clusters.

CN cluster	Sample ID	Age Range*	Sex	Ethnicity/Nationality	Risk factor	Country of origin/birth	Sampling year
CN.01AE.HST/IDU-2	11UK.C2.01	B	Male	Unknown	Heterosexual	South and South East Asia	2011.03
	09UK.C2.02	E	Male	Other Asian/Oriental	IDU	Unknown	2009.04
	11UK.C2.03	B	Unknown	Unknown	Unknown	Unknown	2011.01
	09UK.C2.04	Unknown	Unknown	Unknown	Unknown	Unknown	2009.04
	09UK.C2.05	B	Male	Other Asian/Oriental	Homo/bisexual	South and South East Asia	2009.05
	06UK.C2.06	A	Male	Other Asian/Oriental	Heterosexual	South and South East Asia	2006.04
	09UK.C2.07	C	Male	Other	IDU	Unknown	2009.06
	08UK.C2.08	D	Male	Other Asian/Oriental	IDU	South and South East Asia	2008.09
	06UK.C2.09	C	Female	Other Asian/Oriental	Heterosexual	South and South East Asia	2006.11
	07UK.C2.10	C	Male	Other	Heterosexual	South and South East Asia	2007.01
	08UK.C2.11	C	Male	Other/Mixed	Heterosexual	South and South East Asia	2008.07
	08UK.C2.12	C	Female	Other Asian/Oriental	Heterosexual	South and South East Asia	2008.04
	08UK.C2.13	C	Male	Other Asian/Oriental	other	South and South East Asia	2008.11
	07UK.C2.14	B	Male	Other Asian/Oriental	IDU	South and South East Asia	2007.05
	06UK.C2.15	C	Male	Other Asian/Oriental	IDU	United Kingdom	2006.12
	12UK.C2.16	B	Male	Other Asian/Oriental	Heterosexual	Unknown	2012.01
	07UK.C2.17	C	Female	Other Asian/Oriental	Heterosexual	South and South East Asia	2007.08
	05UK.C2.18	C	Male	Other Asian/Oriental	IDU	South and South East Asia	2005.07
	11UK.C2.19	Unknown	Male	Other Asian/Oriental	Heterosexual	South and South East Asia	2011.06
	05UK.C2.20	D	Unknown	Unknown	Unknown	Unknown	2005.09
	05UK.C2.21	C	Male	Black-other/unspecified	Heterosexual	South and South East Asia	2005.07
	08UK.C2.22	B	Male	Other Asian/Oriental	Homo/bisexual	South and South East Asia	2008.03
	08UK.C2.23	B	Male	Other Asian/Oriental	Heterosexual	South and South East Asia	2010.10
	05UK.C2.24	C	Male	Other/Mixed	Heterosexual	United Kingdom	2005.08
	08UK.C2.25	C	Male	Other/Mixed	Heterosexual	South and South East Asia	2008.05
	06UK.C2.26	Unknown	Male	Other Asian/Oriental	Heterosexual	South and South East Asia	2006.07
	08UK.C2.27	B	Unknown	Unknown	Unknown	Unknown	2008.08
	08UK.C2.28	F	Male	Other	Heterosexual	Caribbean	2008.09
	05UK.C2.29	C	Male	Other Asian/Oriental	IDU	Unknown	2005.02
	05UK.C2.30	D	Male	Black-African	Heterosexual	Southern Africa	2005.06
CN.01AE.MSM-4	10UK.C4.01	D	Male	White	Homo/bisexual	United Kingdom	2010.04
	HUK.C4.02	B	Male	Other Asian/Oriental	Homo/bisexual	East Asia	2011.05
	11UK.C4.03	C	Male	White	Homo/bisexual	Southern Africa	2011.02
	12UK.C4.04	B	Male	Other Asian/Oriental	Homo/bisexual	East Asia	2012.02
CN.01AE.MSM-5	12UK.C5.01	B	Male	White	Homo/bisexual	United Kingdom	2012.06
	11UK.C5.02	D	Male	White	Homo/bisexual	Unknown	2011.08
	12UK.C5.03	B	Male	White	Homo/bisexual	North Africa and Middle East	2012.11
	12UK.C5.04	C	Male	White	Homo/bisexual	United Kingdom	2012.03
	11UK.C5.05	B	Male	Other Asian/Oriental	Homo/bisexual	East Asia	2011.02
	10UK.C5.06	B	Male	White	Homo/bisexual	Unknown	2010.05
	11UK.C5.07	D	Male	White	Heterosexual	United Kingdom	2011.08
	10UK.C5.08	C	Male	Other/Mixed	Heterosexual	East Asia	2010.09
	06UK.C5.09	B	Male	White	Heterosexual	United Kingdom	2006.05
	09UK,C5.10	C	Male	White	Heterosexual	United Kingdom	2009.09
	07UK.C5.11	B	Female	Other/Mixed	Heterosexual	South and South East Asia	2007.09
	09UK.C5.12	E	Male	White	Heterosexual	United Kingdom	2009.08
	09UK.C5.13	F	Male	White	Heterosexual	South and South East Asia	2009.09
	10UK.C5.14	C	Male	White	Heterosexual	United Kingdom	2010.06

*Age Range: A (<20); B (20–29); C (30–39); D (40–49); E (50–59); F (60–69).

**FIGURE 4:**
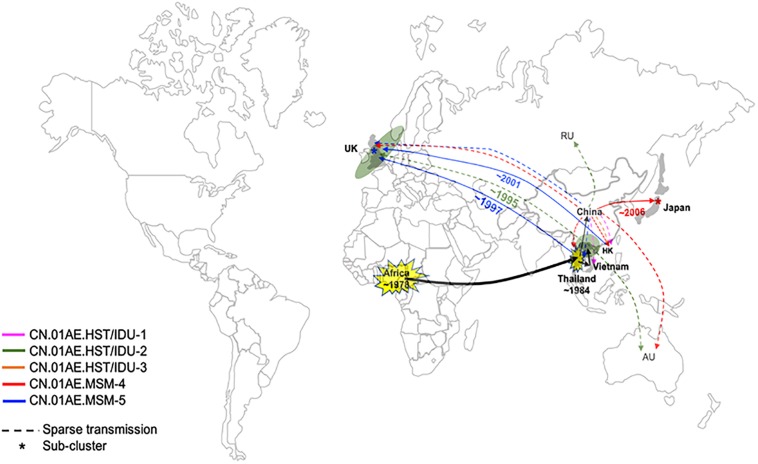
Proposed global dispersal and timeline map of HIV-1 CRF01_AE strains associated with transmission in China. The five CRF01_AE clusters related to HIV transmission in China are color-coded. The color lines represent global dispersal patterns of these different clusters. The black line represents that these related CRF01_AE strains originated from Africa and then spread to Thailand, which became the secondary and the most important source of CRF01_AE, and finally developed several distinct clusters in China. CRF01_AE clusters associated with viral transmission in China have spread to neighboring countries/regions as well as the United Kingdom and other countries. A number of United Kingdom or European sequences and Vietnam sequences were identified within CN.01AE.HST/IDU-2, which was the first out-dispersal cluster and the exportation event occurred around ∼1995. Most CN.01AE.HST/IDU-2 viruses in the United Kingdom were isolated from Asian immigrants. Two distinct sub-clusters within the clusters circulating among Chinese MSM were formed respectively, one of which is in neighboring Japan (designated JP-CN.01AE.MSM.4), and the other is in the United Kingdom with strains from Hong Kong and Thailand (designated UK-CN.01AE.MSM.5).

Interestingly, we found that three United Kingdom MSM sequences, five HK sequences and one Thailand sequence were placed together as another distinct sub-cluster within CN.01AE.MSM-5. The tMRCAs of CN.01AE.MSM-5, the inner node (containing Chinese samples) and the sub-cluster UK-CN.01AE.MSM-5 was 1997, 1998, and 2001, respectively (Figure 3). Similarly, a distinct sub-cluster consisting of Japanese sequences was formed within CN.01AE.MSM-4, with low genetic distances (Figure 2). The molecular clock results showed that the tMRCAs for CN.01AE.MSM-4 and sub-cluster JP-CN.01AE.MSM-4 were 1997 and 2006, respectively (Figure 3). Although previous researchers generally agreed that Japan also played a secondary role in the global epidemic of HIV-1 CRF01_AE strains (Shiino et al., 2014; Angelis et al., 2015), our results indicate that Chinese CRF01_AE strains (CN.01AE.MSM-4) have expanded to Japan around 2006, a little earlier than that reported by Kondo et al. (2013), and possibly caused a potential epidemic in Japan. Of course, some sequences from other countries are also scattered in CN.01AE.MSM-4 and 5. Taken these results together, it suggests that CRF01_AE strains of CN.01AE.MSM-4 and CN.01AE.MSM-5 have been circulating among Chinese MSM for several years, and then dispersed outside, not only to the neighboring countries, but also to the United Kingdom or other countries. Based on our results and previous report (Angelis et al., 2015), we proposed a global migration route map of CRF01_AE clusters circulating in Asia and China (Figure 4).

The major limitations of this study are that (1) only partial sequences (*pol*) were available for this retrospective analysis, (2) the sequences were from only two HIV databases available to this analysis and the sampling of global CRF01_AE strains was less representative, and (3) the sampling periods of the sequences from Los Alamos database and UKHDRD were different, which might influence the investigation of the dispersal patterns.

In conclusion, our analysis suggests that HIV CRF01_AE strains related to viral transmission in China were initially brought to the United Kingdom or other countries by Asian immigrants or international tourists back from Southeast Asia during the 1990s via heterosexuals and IDUs, and then after having circulated among Chinese MSM for several years, they dispersed outside again, possibly through MSM network. This study provided evidence of regional and global dispersal of Chinese CRF01-AE strains. It would also help understand the global landscape of HIV epidemic associated with CRF01-AE transmission and highlight the need for further international collaborative study in this field.

## Data Availability Statement

All datasets generated for this study are included in the article/Supplementary Material.

## Author Contributions

HS and HZ conceived and designed the study. XH and BZ performed data collection. MA performed data analysis and wrote the first draft. HS, HZ, XH, SF, and SE contributed in the process of study design, analyzing data, and writing the manuscript.

## Conflict of Interest

The authors declare that the research was conducted in the absence of any commercial or financial relationships that could be construed as a potential conflict of interest.

## Footnotes

1 www.hiv.lanl.gov

2 www.hivrdb.org.uk

3 http://blast.ncbi.nlm.nih.gov/

## Supplementary Material

FIGURE S1 The reconstructed raw phylogenetic ML tree using all associated sequences after initial BLAST search. Out of 2150 unique sequences from the initial BLAST search, 983 sequences were not clustered with HIV-1 CRF01_AE strains associated with viral transmission in Mainland China. Black branches represent un-clustered sequences from BLAST search, and gray branches represent 42 reference sequences downloaded from the Los Alamos HIV sequence database. The ML tree is rooted with 3 subtype C sequences as outgroup.

TABLE S1 The best fit tree prior for the dataset in Bayesian analysis.

DATA SHEET S1 The alignment of 2150 unique sequences without major drug resistance sites screened after the initial online blast, which was used to reconstruct the phylogenetic tree in this study.

DATA SHEET S2 The raw alignment of 2150 unique sequences after the initial online blast.
