# Supplementary material for: Cross-Continental Dispersal of Major HIV-1 CRF01_AE Clusters in China
Source: Front Microbiol. 2020 Jan 31;11:61. doi: 10.3389/fmicb.2020.00061 (PMC7005055; doi:10.3389/fmicb.2020.00061)
Supplement: FIGURE S1 — The reconstructed raw phylogenetic ML tree using all associated sequences after initial BLAST search. Out of 2150 unique sequences from the initial BLAST search, 983 sequences were not clustered with HIV-1 CRF01_AE strains associated with viral transmission in Mainland China. Black branches represent un-clustered sequences from BLAST search, and gray branches represent 42 reference sequences downloaded from the Los Alamos HIV sequence database. The ML tree is rooted with 3 subtype C sequences as outgroup. [file Presentation_1.PPTX]

## Slide 1
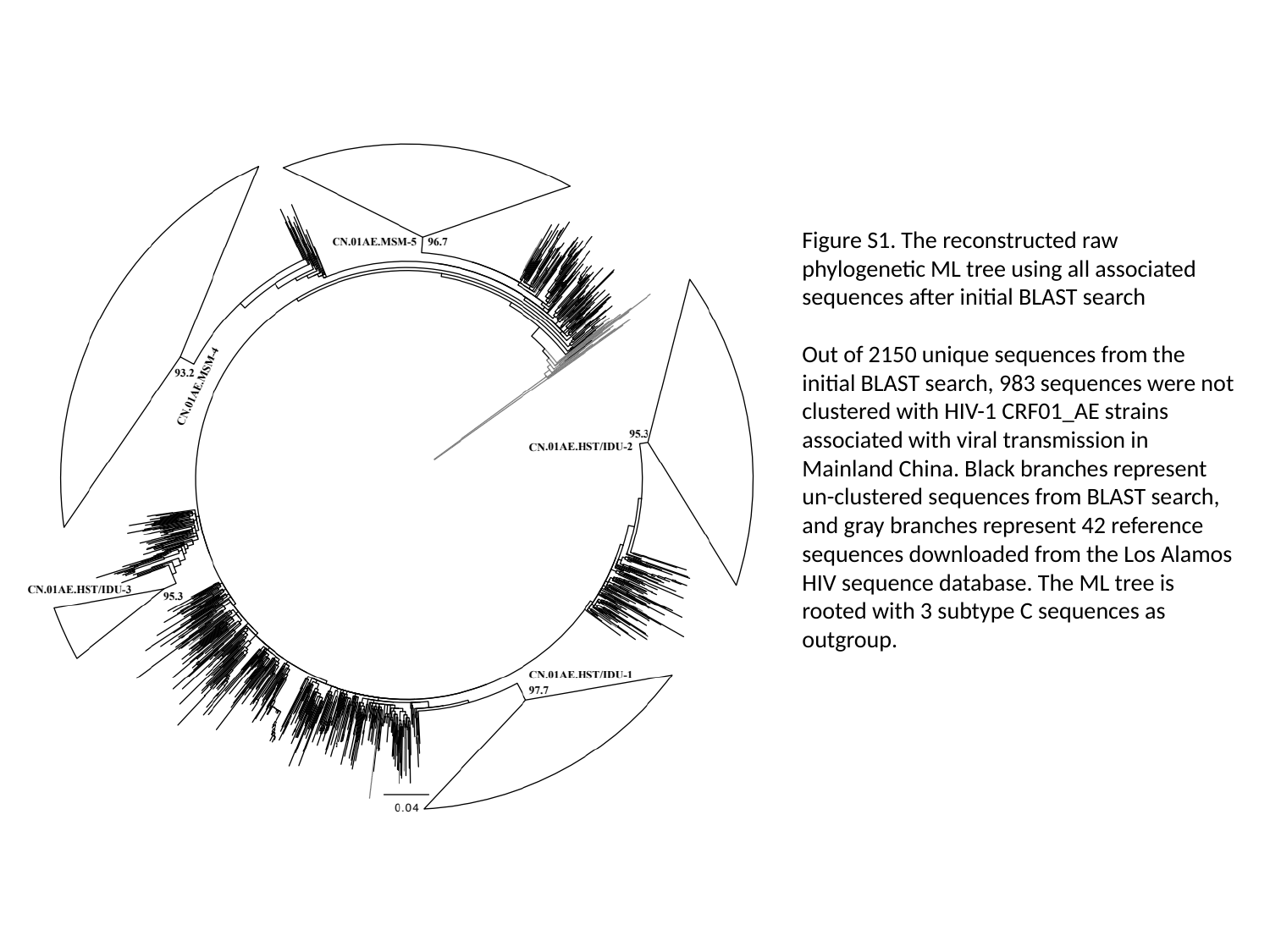

Figure S1. The reconstructed raw phylogenetic ML tree using all associated sequences after initial BLAST search
Out of 2150 unique sequences from the initial BLAST search, 983 sequences were not clustered with HIV-1 CRF01_AE strains associated with viral transmission in Mainland China. Black branches represent un-clustered sequences from BLAST search, and gray branches represent 42 reference sequences downloaded from the Los Alamos HIV sequence database. The ML tree is rooted with 3 subtype C sequences as outgroup.

## Slide 2
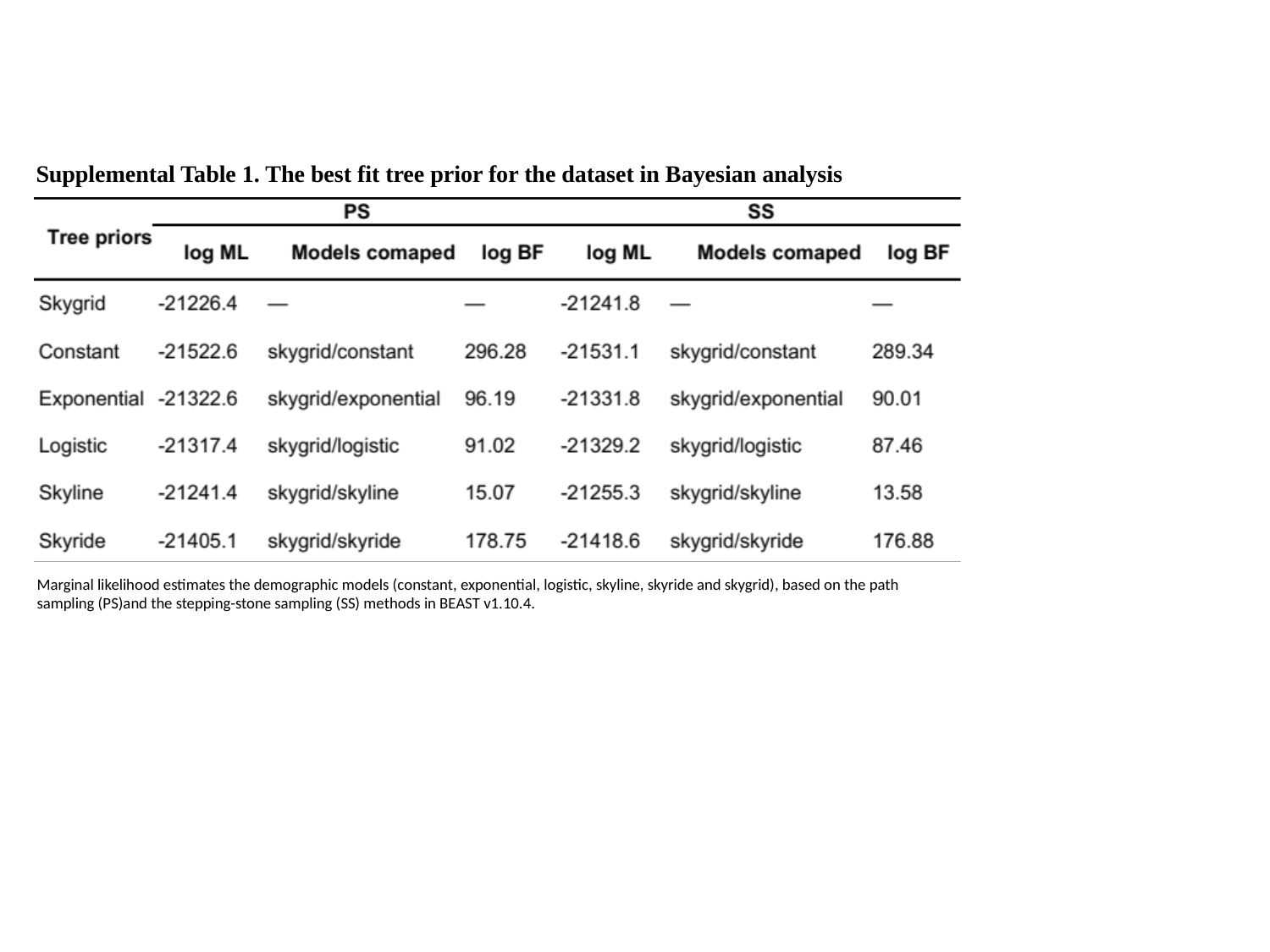

Supplemental Table 1. The best fit tree prior for the dataset in Bayesian analysis
Marginal likelihood estimates the demographic models (constant, exponential, logistic, skyline, skyride and skygrid), based on the path sampling (PS)and the stepping-stone sampling (SS) methods in BEAST v1.10.4.
